# Rhizosphere Microbial Communities Are Significantly Affected by Optimized Phosphorus Management in a Slope Farming System

**DOI:** 10.3389/fmicb.2021.739844

**Published:** 2021-09-13

**Authors:** Qianxin Deng, Tong Zhang, Deti Xie, Yuheng Yang

**Affiliations:** ^1^College of Resources and Environment, Southwest University, Chongqing, China; ^2^College of Plant Protection, Southwest University, Chongqing, China

**Keywords:** phosphorus, rhizosphere microbiota, diversity, community composition, environmental drivers, sloping farmland

## Abstract

Soil rhizosphere microorganisms play crucial roles in promoting plant nutrient absorption and maintaining soil health. However, the effects of different phosphorus (P) managements on soil microbial communities in a slope farming system are poorly understood. Here, rhizosphere microbial communities under two P fertilization levels—conventional (125 kg P_2_O_5_ ha^–1^, P125) and optimal (90 kg P_2_O_5_ ha^–1^, P90)—were compared at four growth stages of maize in a typical sloped farming system. The richness and diversity of rhizosphere bacterial communities showed significant dynamic changes throughout the growth period of maize, while different results were observed in fungal communities. However, both the P fertilization levels and the growth stages influenced the structure and composition of the maize rhizosphere microbiota. Notably, compared to P125, *Pseudomonas*, *Conexibacter*, *Mycobacterium*, *Acidothermus*, Glomeromycota, and *Talaromyces* were significantly enriched in the different growth stages of maize under P90, while the relative abundance of *Fusarium* was significantly decreased during maize harvest. Soil total nitrogen (TN) and pH are the first environmental drivers of change in bacterial and fungal community structures, respectively. The abundance of Gemmatimonadota, Proteobacteria, and Cyanobacteria showed significant correlations with soil TN, while that of Basidiomycota and Mortierellomycota was significantly related to pH. Additionally, P90 strengthened the connection between bacteria, but reduced the links between fungi at the genus level. Our work helps in understanding the role of P fertilization levels in shaping the rhizosphere microbiota and may manipulate beneficial microorganisms for better P use efficiency.

## Introduction

Phosphorus (P) is an essential mineral nutrient for plant growth and development (Wang et al., 2017). Although there generally is P in the soil (Holtan et al., 1988), the available P is usually insufficient for crop production (Luo et al., 2019). The lack of soil P is a main factor limiting crop yield and quality (Heuer et al., 2017) and may even affect global food security (Cordell and White, 2015). Therefore, phosphate fertilizers must be applied to ensure the yield of crops in many crop systems (Salyagiotti et al., 2013; Kaur and Reddy, 2015; Ditta et al., 2018). Many previous studies have shown that plants are not efficient in using phosphate fertilizers (Johri et al., 2015; Batool and Iqbal, 2019), which is due to soil characteristics causing the applied phosphate fertilizer to be transformed into increasingly less soluble forms through various reactions (e.g., precipitation and adsorption) (Withers et al., 2001; Roberts and Johnston, 2015). This may lead to more phosphate fertilizer applications participating in the compounding process in the soil (Neal et al., 2021). Unused phosphate fertilizer by crops is fixed in the soil (Roberts and Johnston, 2015; Luo et al., 2019) or lost from farmland to rivers and lakes through soil erosion, resulting in severe agricultural non-point source pollution (NPSP) (Daverede et al., 2003; Liu et al., 2020). Furthermore, P is a limited and non-renewable resource (Dawson and Hilton, 2011): the raw materials of phosphate fertilizers (rock phosphate) are becoming depleted due to high demands, leading to increases in global fertilizer prices (Lopez-Arredondo et al., 2014; Neal et al., 2021). Integrated P management practices to increase P use efficiency and to reduce environmental pollution are therefore imperative (Roberts and Johnston, 2015).

Most of the P (95–99%) in the soil are phosphate-insoluble compounds and cannot be directly absorbed by plants (Pande et al., 2017). However, many studies have shown that soil microorganisms are indispensable in the soil P cycle and therefore play pivotal roles in improving the absorption of P by plants (Richardson, 2001; Rashid et al., 2016; Anzuay et al., 2017; Ceci et al., 2018). Soil microorganisms (bacteria and fungi) can convert insoluble forms of P into an accessible form that can be absorbed by plants (Paul et al., 2018). These kinds of microorganisms are called phosphate-solubilizing microorganisms (PSMs), which include phosphate-solubilizing bacteria (PSB) and phosphate-solubilizing fungi (PSF) (Qian et al., 2019). The PSB involved in the phosphorus conversion process are *Pseudomonas*, *Mycobacterium*, *Bacillus subtilis*, *Rhizobium*, *Azotobacter*, and *Agrobacterium* (Babalola and Glick, 2012; Sharma et al., 2013; Qian et al., 2019). Some studies have shown that PSB may decrease the pH of the soil through many processes in order to promote the solubilization of soil P (Adnan et al., 2017), such as releasing organic acids and hydrogen ions (Xiao et al., 2017) and producing siderophores and phosphatases (Rodriguez and Fraga, 1999). The most common PSF are *Aspergillus* and *Penicillium* (Seshadri et al., 2004; Rashid et al., 2016). Other species such as *Talaromyces* and *Eupenicillium* also play important roles in the P cycle (Della Monica et al., 2014).

Several reports have shown that changes in soil rhizosphere microbial communities are largely affected by agricultural management practices (Hartmann et al., 2015; Li et al., 2015a; Tian et al., 2015), and the response of microbial communities to phosphate fertilizer input varies significantly in different regions and ecosystems (Huang et al., 2016; Long et al., 2018; Wang et al., 2018). For example, the P input rates (0–32 g P_2_O_5_ m^–2^ year^–1^) were negatively correlated to the richness of soil bacterial communities in a semi-arid steppe (Ling et al., 2017), while a high P input (30 g P_2_O_5_ m^–2^ year^–1^) in forest soil increased the abundance of soil microbial communities (Huang et al., 2016). Moreover, a low P input (50 mg P_2_O_5_ kg^–1^) increased the diversity of endophytic bacteria and a high P input (200 mg P_2_O_5_ kg^–1^) increased the diversity of fungi in P-deficient paddy soils (Long and Yao, 2020). In contrast, excessive P input (131 kg P ha^–1^) reduced the diversity of maize arbuscular mycorrhiza (AM) in the North China Plain (Lang et al., 2018). However, these studies were carried out at a single time point. Few studies have been carried out on maize sloping farming systems where P is easily lost due to water erosion (surface runoff) (Strehmel et al., 2016; Zhang et al., 2017).

Our previous results showed that optimized P management had a positive effect on maize yield (Zhang Y. et al., 2019), while the interaction mechanism between phosphate fertilizer–soil rhizosphere microorganisms–plants is unknown. Thus, we performed a study with temporal resolution to quantitatively understand how the maize soil rhizosphere microorganisms of sloping farmlands are affected by phosphate fertilization (P125 and P90). We hypothesized that P90 would lead to a significant enrichment of some microorganisms involved in the P cycle that can contribute to P availability in sloping farmlands. For this purpose, we collected soil samples in four growth stages of maize and investigated the responses of rhizosphere bacteria and fungi to different P supply levels using high-throughput sequencing. Our work helps in understanding the changing process of the rhizosphere microbiota during the entire growth cycle of maize under different P levels and provides theoretical support for the efficient use of P in the rhizosphere.

## Materials and Methods

### Study Area and Experimental Design

Our study area is located in the hinterland of the Three Gorges Reservoir Area (TGRA) in China (29°30′ N, 107°18′ E), where more than 60% of the lands are sloped and over 70% of the soil has suffered from serious soil erosion (Zhang T. et al., 2019). The typical tillage practice is a maize–mustard rotation in the sloping farmland of the TGRA (Zhang Y. et al., 2019). The annual average temperature of the study area is 22.1°C, the monthly average maximum temperature is 30°C, the monthly average minimum temperature is 8°C, and the average annual rainfall is 1,130 mm (Zhang et al., 2020). At the beginning of the experiment, the soil had a pH of 5.34, total nitrogen (N) of 0.77 g kg^–1^, available P of 24.2 mg kg^–1^, nitrate N (NO_3_–N) of 35.8 mg kg^–1^, and ammonium N (NH_4_–N) of 31.6 mg kg^–1^ (Zhang Y. et al., 2019).

Based on a previous report by our group, reducing the amount of conventional fertilization by one-quarter to one-half could not affect crop nutrient absorption and also reduced soil nutrient loss (Zhang Y. et al., 2019). We initiated the field experiment on March 5, 2019, and included the following two phosphate fertilizer (as superphosphate) treatments with three replicated plots (100 m^2^ per plot): (1) conventional fertilization (125 kg P_2_O_5_ ha^–1^, P125) and (2) optimal fertilization (90 kg P_2_O_5_ ha^–1^, P90). The application rates of N fertilizer (as urea) and potash (K) fertilizer were the same as those of the traditional local application rates: 343 kg N ha^–1^ and 210 kg KCl ha^–1^, respectively. Two-thirds of the N and K fertilizers and four-fifths of P were supplied as basal fertilizers on March 25, 2019. The others were applied for top dressing on May 4, 2019. The slopes of plots were measured for an average of 12°. An impermeable high-density waterproof polyethylene board was embedded into the soil between each studied plot. It was used to avoid the mutual influence among treatments by preventing lateral and transverse migrations of nutrients and water between plots.

### Soil Sampling and Biogeochemical Analysis

At the maize seedling stage [emergence to the second leaf stage (S), April 17, 2019], ear stage [second leaf to tassel (E), May 26, 2019], flowering stage [silking to milk stage (F), July 2, 2019], and harvest stage [milk to maturity (H), August 5, 2019], a total of 24 rhizosphere soil samples were collected at depths of 10–30 cm. In each repeated plot, five complete maize roots were dug out according to the five-point sampling method; the large blocks around the roots were shaken off. Then, the soil on the surface of the root system was gently brushed down and the rhizosphere soil was obtained. After three repetitions for each treatment, the collected samples were immediately transported on ice to the laboratory. Part of the air-dried soil samples was used for analysis of soil physical–chemical properties, while the other samples were used to extract total soil DNA. During the H stage, we further calculated the weight of the maize grain and straw for subsequent yield analysis.

Soil physiochemical analysis methods were used according to a previous study (Ali et al., 2019). The soil pH was measured in water (1:2.5 soil/water) with a glass electrode. Total N (TN) was determined using the Kjeldahl method. Available N (AN) was measured with the alkaline solution diffusion method. Available P (AP) was extracted with NaHCO_3_ and determined using the ammonium molybdate–antimony potassium tartrate-ascorbic acid method. Soil NO_3_–N and NH_4_–N were extracted with 0.01 mol L^–1^ calcium chloride and determined with a continuous flow analyzer (AA3, SEAL Company, Norderstedt, Germany).

### DNA Extraction, PCR Amplification, and Metagenomic Sequencing

For each sample, soil microbial DNA (24 in total) was extracted from approximately 0.5 g of frozen soil using a MoBio PowerSoil DNA Isolation Kit (MoBio Laboratories, Carlsbad, CA, United States) according to the manufacturer’s protocols. The final DNA concentration and purification were determined by NanoDrop 2000 UV–Vis spectrophotometer (Thermo Scientific, Wilmington, DE, United States), and the DNA quality was checked with 1% agarose gel electrophoresis. Distinct regions of bacterial 16S ribosomal RNA (rRNA) and fungal internal transcribed spacer (ITS) genes were amplified using specific primers (Supplementary Table 1) *via* a thermocycler PCR system (GeneAmp 9700, Applied Biosystems, Frederick, MD, United States). The PCR amplification processes of the 16S rRNA gene and the ITS gene are shown in Supplementary Table 2. Amplicons were extracted from 2% agarose gels and further purified using the AxyPrep DNA Gel Extraction Kit (Axygen Biosciences, Union City, CA, United States), then quantified using QuantiFluor^TM^-ST (Promega, Madison, WI, United States) according to the manufacturer’s protocol. The purified amplicons were pooled in equimolar and paired-end sequenced on an Illumina MiSeq PE300 platform (Illumina, San Diego, CA, United States) according to the standard protocols by Majorbio Bio-Pharm Technology Co. Ltd. (Shanghai, China).

### Data Analysis

Raw fastq files were demultiplexed, quality-filtered by fastp version 0.20.0 (Chen et al., 2018), and merged by FLASH version 1.2.11 (Magoc and Salzberg, 2011) with the following criteria: (i) reads were truncated at any site receiving an average quality score <20 over a 50-bp sliding window; (ii) primers were exactly matched allowing two nucleotides mismatching and reads containing ambiguous bases were removed; and (iii) sequences whose overlap was longer than 10 bp were merged according to their overlap sequence. Operational taxonomic units (OTUs) were clustered with a 97% similarity cutoff using UPARSE 7.1^1^ (Edgar, 2013), and chimeric sequences were identified and removed using UCHIME. The taxonomy of each 16S rRNA and ITS gene sequence was analyzed using the RDP Classifier algorithm^2^ (Wang et al., 2007) against the 16S rRNA database (Silva v.132) and the ITS database (UNITE v.8.0) using a confidence threshold of 70%. This study uses the current taxonomy terminology (e.g., Actinobacteria now “Actinobacteriota,” Acidobacteria now “Acidobacteriota,” Bacteroidetes now “Bacteroidota,” etc.) (Parks et al., 2018).

### Statistical Analysis

Before the analysis, we rarefied all sequences based on the minimum sequence number (35,559 for bacterium and 34,497 for fungus) to correct for differences in sequencing depths. We have taken the rank of OTU as the abscissa and the number of sequences contained in each OTU as the ordinate to draw the graph using R language. The Chao and Shannon indexes were used to evaluate the alpha diversity of the microorganisms (Mothur 1.30.2). A principal coordinate analysis (PCoA) was constructed based on the Bray–Curtis dissimilarity matrix and weighted UniFrac matrix to visualize the differences in the microbial composition (ANOSIM in R language with 999 permutations) (Liu et al., 2020). The compositions of the microbial community under the two treatments were analyzed with R language. One-way ANOVA and Welch’s *t*-test were used to discover species with significant differences between groups (*p* < 0.05), and *p*-values were multiple corrected by false discovery rate (FDR). Redundancy analysis (RDA) was applied to reflect the relationship between the environmental factors and the rhizosphere microbial community composition (vegan package in R). Heatmaps based on Spearman’s coefficients were used to show specific microorganisms related to environmental factors. In addition, bacterial and fungi genera with average relative abundance in the top 100 of two treatments were selected for microbial interaction network analysis (using the networkx tool). Then, four microbial networks were built based on a strong correlation (Spearman’s correlation coefficient of >0.5) and significance (*p* < 0.05). The topological properties of these networks were estimated with the networkx tool.

## Results

### Soil Biogeochemical Properties and Crop Yields

Different P fertilization treatments caused significant differences (*p* < 0.05) in rhizosphere soil pH, AN, AP, TN, NO_3_–N, and NH_4_–N at four growth periods of maize (Table 1). Compared with P125, P90 resulted in significant reductions in soil pH (entire growth period), TN (E and F), AN, and NH_4_–N (E, F, and H). AP and NO_3_–N had no obvious regularity in the different growth stages. In addition, two yield indicators (grain and straw) showed that P90 significantly increased maize yield (*p* < 0.05; Supplementary Figure 1).

**TABLE 1:** Soil biogeochemical properties in the four growth stages of maize—seedling stage (S), ear stage (E), flowering stage (F), and harvest stage (H)—under conventional treatment (P125, rate of 125 kg P_2_O_5_ ha^–1^) and optimal treatment (P90, rate of 90 kg P_2_O_5_ ha^–1^).

Soil properties		Growth periods			
		S	E	F	H
pH (1:2.5 H_2_O)	P125	5.37 ± 0.01 a	5.39 ± 0.01 a	5.43 ± 0.01 a	5.38 ± 0.00 a
	P90	5.33 ± 0.00 b	5.35 ± 0.01 b	5.38 ± 0.01 b	5.35 ± 0.01 b
Available N (mg kg^–1^)	P125	189.19 ± 0.63 a	208.96 ± 0.99 a	221.54 ± 0.49 a	186.29 ± 0.54 a
	P90	189.24 ± 0.53 a	195.05 ± 0.34 b	218.38 ± 0.35 b	178.03 ± 0.67 b
Available P (mg kg^–1^)	P125	17.18 ± 0.32 b	21.07 ± 0.59 a	11.47 ± 0.38 b	26.39 ± 0.54 a
	P90	20.20 ± 0.44 a	16.42 ± 0.31 b	13.23 ± 0.27 a	23.06 ± 0.77 b
Total nitrogen (mg kg^–1^)	P125	1.08 ± 0.01 a	1.19 ± 0.02 a	1.12 ± 0.01 a	1.09 ± 0.01 a
	P90	1.06 ± 0.01 a	1.06 ± 0.01 b	1.06 ± 0.01 b	1.08 ± 0.01 a
Nitrate N (mg kg^–1^)	P125	35.31 ± 0.49 a	29.27 ± 0.36 b	34.44 ± 0.86 a	26.24 ± 0.59 a
	P90	33.59 ± 0.44 b	38.07 ± 0.13 a	34.54 ± 0.20 a	23.15 ± 0.23 b
Ammonium N (mg kg^–1^)	P125	20.53 ± 0.38 a	25.33 ± 0.27 a	30.49 ± 0.54 a	17.54 ± 0.51 a
	P90T	20.66 ± 0.16 a	24.35 ± 0.30 b	29.42 ± 0.31 b	15.41 ± 0.28 b

*Different letters in the same column indicate significant differences between two treatments at the same growth stage (*p* < 0.05).*

### Taxonomic Characteristics, Diversity, and Richness Index of Microbial Communities

For the entire sampling set, a total of 1,389,235 bacterial sequences and 1,514,552 fungal sequences were quality filtered, chimera checked, and obtained with average lengths of 255 and 244 bp. These classified sequences were further used to cluster the OTUs at a 3% dissimilarity level. A set of 4,631 bacterial OTUs and 1,585 fungal OTUs was obtained, and species taxonomic analysis was also computed. All the sampling efforts tended to reach the saturation plateau in the rarefaction analysis and were effective in covering the full extent of almost a majority of the bacterial and fungal diversity according to a 97% sequence similarity in the rank abundance curve approach (Supplementary Figure 2).

**FIGURE 1:**
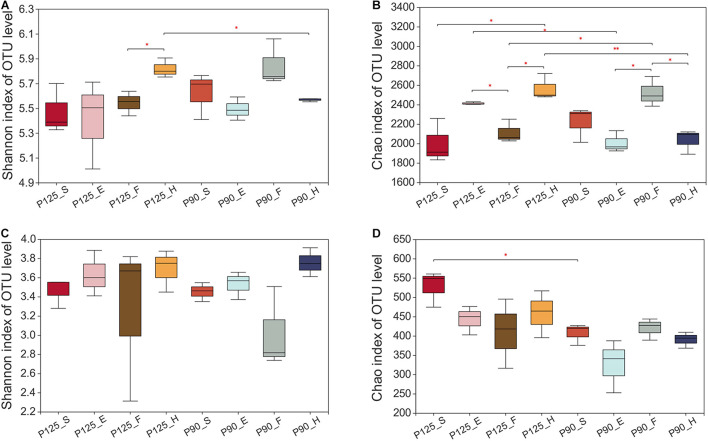
Alpha diversity of the bacterial and fungal communities in the four growth stages of maize—seedling stage (*S*), ear stage (*E*), flowering stage (*F*), and harvest stage (*H*)—under conventional treatment (P125, with a rate of 125 kg P_2_O_5_ ha^–1^) and optimal treatment (P90, with a rate of 90 kg P_2_O_5_ ha^–1^). **(A,B)** Shannon diversity index **(A)** and Chao richness index **(B)** of the bacterial community. **(C,D)** Shannon diversity index **(C)** and Chao richness index **(D)** of the fungal community. Significant differences between the same growth periods under two treatments were compared using Welch’s *t*-test. ^∗^0.01 < *p* ≤ 0.05; ^∗∗^0.001 < *p* ≤ 0.01.

**FIGURE 2:**
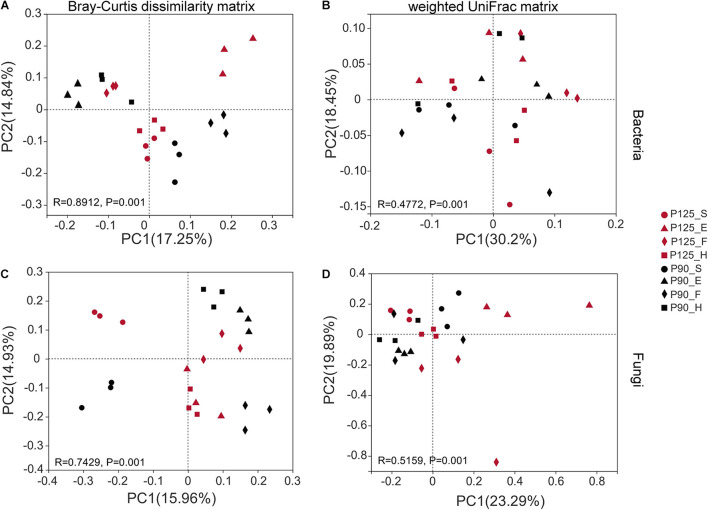
Beta diversity of the microbial community was analyzed using the principal coordinate analysis (PCoA) diagram based on the Bray–Curtis dissimilarity matrix and weighted UniFrac matrix. **(A,B)** Bacterial community. **(C,D)** Fungal community. The percentage of the two main axes represents the explanatory value of the difference in the sample composition. *Points of different colors* and *shapes* represent different treatments (P125, with a rate of 125 kg P_2_O_5_ ha^–1^; P90, with a rate of 90 kg P_2_O_5_ ha^–1^) and growth stages [seedling stage (*S*), ear stage (*E*), flowering stage (*F*), and harvest stage (*H*)], respectively. The distance between two sample points indicates the similarity of the species composition of the two samples (*p* = 0.001).

Maize has unique rhizosphere microbiota in different growth periods and P treatments. Under P125, the bacterial Shannon diversity index was significantly higher in the H stage compared with that in the F stage, and the Chao index was significantly lower in the F stage compared with that in the E and H stages (*p* < 0.05; Figures 1A,B). However, under P90, the Chao index was significantly higher in the F stage than that in the E and H stages (*p* < 0.05; Figure 1B). There was no significant difference in the Shannon index between the various growth stages of fungus, and P90 significantly decreased the richness of fungus at the S stage (Figures 2C,D). Meanwhile, P90 significantly decreased the diversity and richness of bacteria at the H stage. P125 significantly decreased the richness of bacteria at the F stage and increased the Chao index of the E stage (*p* < 0.05; Figure 1B). For a comparison of the differences in the structure of the rhizosphere microbiota, PCoA of the Bray–Curtis distance and weighted UniFrac matrices were performed. The results indicated that the rhizosphere microbial community structure of bacteria in each growth period formed separated clusters of varying degrees in the first two coordinate axes (*R* = 0.8912 for the Bray–Curtis distance and *R* = 0.4772 for the weighted UniFrac matrices) (Figures 2A,B), and similar results for fungi were also observed (Figures 2C,D). It is worth noting that there was no obvious separation of the bacterial communities in the maize S stage, while a distinct separation was observed in fungal communities. All these results showed that the rhizosphere microbial community structures were changed with the growth of maize and the P fertilization treatments.

### Changes in Microbial Community Composition and Structure

**FIGURE 3:**
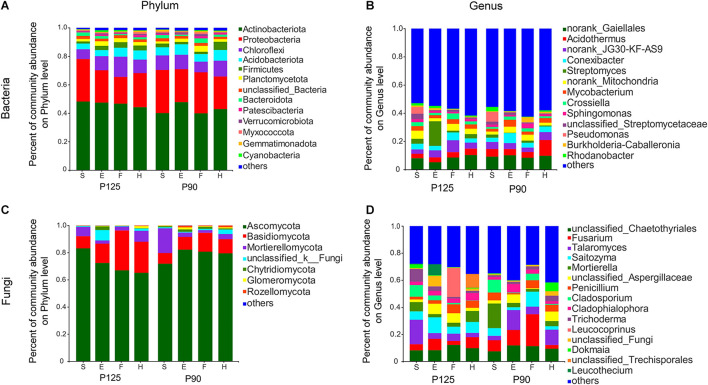
Community composition of bacteria and fungi in four growth stages—seedling stage (*S*), ear stage (*E*), flowering stage (*F*), and harvest stage (*H*)—under conventional treatment (P125, with a rate of 125 kg P_2_O_5_ ha^–1^) and optimal treatment (P90, with a rate of 90 kg P_2_O_5_ ha^–1^). **(A,B)** Bacterial community composition at the phylum and genus levels. **(C,D)** Fungal community composition at the phylum and genus levels. *Columns of different colors* represent different species; the *length of the columns* represents the proportion of the species.

To further study the effect of P treatment on rhizosphere taxa in the entire maize life cycle, we compared the relative abundance of rhizosphere microbiota at the phylum and genus levels. Although the dominant microbiota from the two treatments were largely consistent over time (Figure 3), several specific rhizosphere taxa changed throughout the growth cycle or single growth period of maize in the two treatments. For bacteria, P90 significantly increased the relative abundance of four phyla and five genera and decreased the abundance of three phyla and three genera during the various growth stages of maize (*p* < 0.05; Supplementary Figures 3A,B). Moreover, the rhizosphere microbiota of P125 and P90 showed obvious differences at the phylum and genus levels across the entire growth stage (*p* < 0.05; Supplementary Figure 5). The relative abundance of *Streptomyces* and *Crossiella* showed significant differences in P125, whereas *Acidothermus*, *Conexibacter*, *Mycobacterium*, and *Pseudomonas* dramatically changed in P90 (*p* < 0.05; Supplementary Figures 5C,D). For fungi, Glomeromycota significantly increased during the F stage only in P90 (*p* < 0.05; Supplementary Figure 4A). P90 significantly decreased the abundance of *Fusarium*, *Leucothecium*, and *Cladophialophora* during the E and H stages, while it increased the abundance of *Talaromyces* and *Knufia* (*p* < 0.05; Supplementary Figure 4B). Furthermore, the relative abundance of Mortierellomycota, *Mortierella*, and *Cladosporium* in P125 showed large differences at both the phylum and genus levels throughout the whole growth period, but no difference was observed in P90 at the phylum level (*p* < 0.05; Supplementary Figure 6).

### Microbial Community Structures and Their Relationships With Soil Properties

**FIGURE 4:**
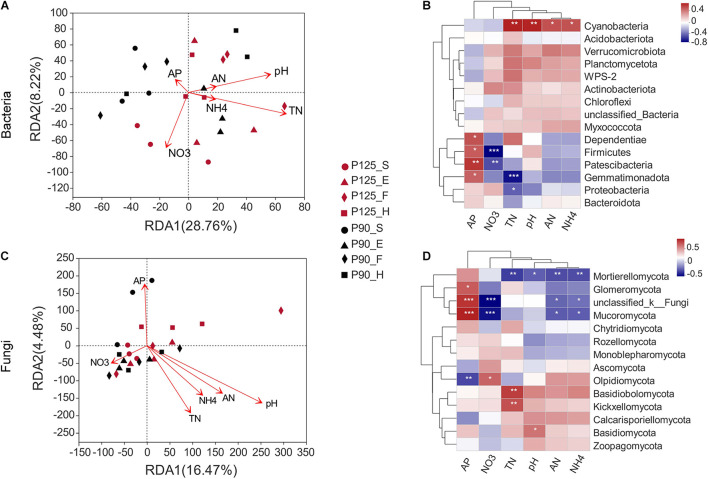
Correlation of soil microbial community structure–environmental factors–specific microorganisms by redundancy analysis (RDA) and heatmaps. **(A,B)** Bacterial community structure. **(C,D)** Fungal community structure. Soil properties included available P (*AP*), pH, available N (AN), total N (TN), ammonium N (NH_4_), and nitrate N (NO_3_). The *direction of the arrow* represents the correlation with the first two-gage axes; the *length of the arrow* indicates the strength of the correlation. *Colors in the heatmap* indicate different correlation thresholds (*R*), and the color interval is shown in the *upper right* legend. *P*-values are used to express significance. *0.01 < *p* ≤ 0.05; **0.001 < *p* ≤ 0.01; ***p ≤ 0.001. *P125*, treatment of 125 kg P_2_O_5_ ha^–1^; *P90*, treatment of 90 kg P_2_O_5_ ha^–1^; *S*, seedling stage; *E*, ear stage; *F*, flowering stage; *H*, harvest stage.

The RDA indicated obvious relationships between soil properties and microbial community composition. The first and second axes show that 28.76 and 8.22% of the changes in the bacterial community were influenced by soil properties (Figure 4A). The RDA results showed a significant correlation of soil TN with bacterial community structures (*p* = 0.025; Supplementary Table 3). The abundance of Gemmatimonadota, Proteobacteria, and Cyanobacteria was associated with soil TN (*p* < 0.05; Figure 4B). Moreover, soil pH significantly affected the fungal community structures (*p* = 0.003; Figure 4C and Supplementary Table 3). Simultaneously, the slight change in pH level was related to the abundance of Basidiomycota and Mortierellomycota (*p* < 0.05; Figure 4D).

### Microbial Networks

**FIGURE 5:**
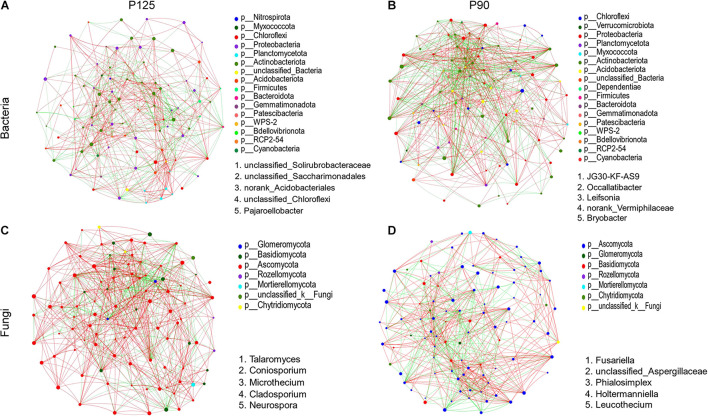
**(A–D)** Network analysis showing the connectedness among genera of bacteria or fungi in the soils under P125 **(A,C)** and P90 **(B,D)**. Nodes (*colored dots*) imply the genera involved in the networks, and the *size of the node* indicates the abundance of species. *Links* mean the relationship among the nodes: *Red* and *green lines* represent positive and negative correlations, respectively. *Dots of different colors* indicate the different phyla to which the genera belong. The keystone genera are based on the top five betweenness centrality scores in the network.

At the genus level, microbial networks were established based on correlations for the soil samples in P125 and P90 (Figure 5). The networks of bacteria consisted of 100 (P125) and 97 (P90) nodes (Figures 5A,B and Supplementary Table 4). The networks of fungus captured 99 nodes in each treatment (Figures 5C,D and Supplementary Table 5). For bacteria, the degree and clustering coefficients were much higher in P90 than those in P125 (Supplementary Table 4). The average shortest path length and the degree and clustering coefficients of fungus in P90 networks were much lower than those in P125 (Supplementary Table 5). Additionally, the keystone taxa based on the betweenness centrality scores also varied in P90 and P125 for the microbial networks of bacteria and fungus (Figure 5 and Supplementary Tables 4, 5).

## Discussion

Previous studies have demonstrated that the input of agricultural P into the Yangtze River increased from 55 to 81% with extensive use of chemical fertilizers (Liu X.C. et al., 2018). In addition, non-point-source P comes mainly from the surface runoff of sloped farming in the TGRA (Zhu et al., 2012). Therefore, optimizing fertilization management and utilizing beneficial microorganisms on sloping croplands are essential for reducing fertilizer consumption and controlling NPSP in this region.

### Effects of P Fertilization on the Bacterial Community in Different Growth Periods of Maize

Plant roots lead to the selective enrichment of rhizosphere microorganisms due to environmental changes (Berendsen et al., 2012; Lareen et al., 2016). Previous work have shown that P levels would affect bacterial communities under different soil types, plant species, and crop growth periods (Huang et al., 2016; Lang et al., 2018; Long et al., 2018; Wang et al., 2018; Zhuang et al., 2020). We performed a longitudinal comparison of the changes across the entire life cycle of maize in two treatments. Dynamic changes in the diversity and richness of the bacterial communities were observed between different growth periods (Figures 1A,B). Subsequently, the maize rhizosphere microbiota under two treatments shifted in different developmental stages (Figures 2A,B, 3A,B), showing that plants may recruit different rhizosphere microorganisms at different growth stages and conditions. For example, under P90, *Pseudomonas*, *Conexibacter*, and *Mycobacterium* were significantly enriched in the S and E stages (Supplementary Figure 5D), indicating that maize may actively recruit these beneficial bacteria to participate in the P cycle and promote its rapid growth. The dynamic changes of the rhizosphere microbiota throughout the maize life cycle may be due to the changes in the structure of the root system and the release of root exudates. In the past, some studies have shown that the bacterial community structure varied with the developmental stages of maize, and these results were explained as a sign of the complex interplay between microbes and root exudates (Baudoin et al., 2003; Guo et al., 2017; Hu et al., 2018).

### Effects of P Fertilization on the Fungal Community in Different Growth Periods of Maize

Previous studies have shown that P fertilizer affects the diversity and community structure of fungi, but these works were carried out at a single time point only or under different soil types (Fanin et al., 2015; Luo et al., 2015; Huang et al., 2016; Lang et al., 2018; Liu M. et al., 2018). Our research results demonstrated that there were no significant differences in the Shannon and Chao indexes of fungi (Figures 1C,D), while distinct changes in the community composition of fungi were observed (Figures 2C,D). Interestingly, the fungal communities under two P treatments showed significant differences in the S stage compared to bacteria (Figure 2C), indicating that bacteria are not as sensitive to P addition as fungi in the early stage of maize growth. This result paralleled recent studies showing that the fungal hyphae of plant roots could rely on their longer hyphae length to increase the nutrient absorption area, which may have contributed to the sensitivity of fungi to P addition (Li et al., 2015b; Liu M. et al., 2018). Moreover, this conclusion was supported by another result of our research whereby P90 increased the relative abundance of the fungal phylum Glomeromycota in the first three growth stages of maize, especially at the F stage (Supplementary Figures 4A, 6A,B). It is well accepted that AM fungi can facilitate the absorption of soil P by plants, and all AM fungi are members of the phylum Glomeromycota, which can coexist with 70–90% of vascular plants in the world (Smith et al., 2011; Liu et al., 2012; Sangabriel-Conde et al., 2015; Crossay et al., 2018; Sturmer et al., 2018). It is interesting to note that the relative abundance of *Fusarium* under P90 was significantly reduced during the H stage compared to that under P125 (Supplementary Figure 4B). We reasoned that the significant increase in the relative abundance of *Pseudomonas* in the S stage under P90 resulted in a decrease in the relative abundance of *Fusarium* (Supplementary Figure 5D). Previous studies have shown that antifungal compounds from *Pseudomonas* are strongly antagonistic to *Fusarium* (Pal et al., 2001; Figueroa-Lopez et al., 2016). In addition, the increase in the relative abundance of Glomeromycota may also be involved in the competition for space and nutrients with pathogenic microorganisms (Martinez-Viveros et al., 2010; Olowe et al., 2018).

### Relationship Between Soil Microbial Community and Soil Properties

Previous studies have shown that changes in the P content affect the soil microbial community structure (DeForest and Scott, 2010; Luo et al., 2015). However, other observations indicated that other soil properties such as pH, TN, AN, and phosphatase were also involved in the process of shaping the structure of soil microbial communities (Luo et al., 2015; Shen et al., 2015; Kaiser et al., 2016; Ai et al., 2018; Neal et al., 2021). In our study, soil TN has a significant correlation with the bacterial community structure, which was affected by P fertilization (Figure 4A and Supplementary Table 3). This observation is in line with previous studies whereby P fertilization increased TN and that variables related to N conversion are important factors that determine the structure of bacterial communities in soils with other similar nutrient contents (Williams et al., 2013; Shen et al., 2015). We also found that changes in soil TN correlated with Gemmatimonadota, Proteobacteria, and Cyanobacteria, which was supported by the results of recent studies reporting that Gemmatimonadota participated in the process of soil N cycle (Bhatti et al., 2017). Furthermore, the fungal structure was significantly correlated with soil pH, unlike the bacterial community structure (Figure 4C and Supplementary Table 3). Previous results have shown that the effects of P addition on fungal communities were mainly through its indirect influence on soil pH (Wakelin et al., 2009, 2012; Huang et al., 2016; Ai et al., 2018). In our study, the pH in P125 was higher than that in P90, which agrees with previous studies suggesting that P addition increases soil basic cations by stimulating soil microbial nutrient conversion and eventually elevating soil pH (Liu et al., 2013; Zhu et al., 2015). Additionally, the abundance of Basidiomycota and Mortierellomycota was associated with soil pH and might have resulted from P fertilization. Altogether, these results showed that soil TN and pH might be the crucial factors affecting the soil rhizosphere community structure in purple soil sloping farmland.

### Differences in P Fertilization Levels Affect the Interactions Between Microorganisms

Network analysis can determine the interactions between microbial groups in niches and identify the keystone taxa most closely related to the microbial community (Liu et al., 2013; Vick-Majors et al., 2014; Banerjee et al., 2016). In the networks acquired for the soil bacteria, the correlations between genera and degrees were both increased in P90 compared to those in P125 (Figures 5A,B). Thus, the soil bacterial communities in P90 formed more complex networks, which suggests that a strong network complexity could increase the stability of the rhizosphere microbial community structure (Mander et al., 2012; Zheng et al., 2018). For the microbial networks of fungi, P90 reduced the interactions between microorganisms in the network, which shows that a higher P input (P125) might shape a more stable microbial network (Figures 5C,D). Considering the differences in the diversity and richness of the fungal communities under the two treatments, it is likely that higher diversity and richness of species might increase the complexity of the network and strengthen the interactions between microorganisms.

Moreover, the keystone genera of the bacterial and fungal communities were also different under the two treatments (Figure 5 and Supplementary Tables 4, 5). Although their relative abundance in the microbial community was lower, they can exert a disproportionately large effect in the soil ecosystem (Zheng et al., 2018). For bacterial communities, the keystone genera in P90 are *Leifsonia* and *Bryobacter*. According to recent reports, *Leifsonia* is a mesophilic species commonly found in weakly acidic soil; it can affect the bioavailability of P and other mineral nutrients in the soil (Tan et al., 2020). In addition, *Bryobacter* could utilize polysaccharides, various sugars, and organic acids, with a significant impact on the biogeochemical carbon cycle (Dedysh et al., 2017; Liu et al., 2019). For the fungal communities, *Talaromyces* was identified as a keystone genus in P125. *Talaromyces* showed desired P-solubilizing capability in recalcitrant phosphate, which was widely reported in recent studies (Mendes et al., 2014; Zhang et al., 2018). In contrast, *Fusariella* was the keystone genus in P90, which might explain the lower stability of the fungal community structure in this treatment level (Fu et al., 2017; Dugdale et al., 2018). These results suggest that different P input levels might affect the stability of the entire community by affecting certain specific species of the rhizosphere microbial community.

## Conclusion

Our research showed that the diversity and richness of the bacterial and fungal communities during the growth cycle of maize respond differently to the two P input levels. The rhizosphere microbial community structure was strongly influenced by P fertilization levels and growth stages and was significantly related to soil TN and pH. Maize could recruit different rhizosphere microorganisms, such as *Pseudomonas*, *Conexibacter*, *Mycobacterium*, *Acidothermus*, Glomeromycota, and *Talaromyces*, in order to adapt to changes in the soil environment and promote rapid crop growth. Moreover, P90 enhanced the interaction among the genera in the microbial network of bacteria, while it reduced the stability of the fungal community. Further research should be conducted to investigate the process and mechanism of the effects of different phosphate fertilizer levels on bacteria and fungi.

## Data Availability Statement

The datasets presented in this study can be found in online repositories. The names of the repository/repositories and accession number(s) can be found in the article/Supplementary Material.

## Author Contributions

All authors have contributed to the intellectual input and assistance to this study and to manuscript preparation. TZ conceived and designed the study. QD and YY collected the soil samples. QD did the experiments and performed the data analysis, with help from TZ and YY. QD wrote the manuscript. DX, TZ, and YY revised the manuscript. All authors read and approved the final manuscript.

## Conflict of Interest

The authors declare that the research was conducted in the absence of any commercial or financial relationships that could be construed as a potential conflict of interest.

## Publisher’s Note

All claims expressed in this article are solely those of the authors and do not necessarily represent those of their affiliated organizations, or those of the publisher, the editors and the reviewers. Any product that may be evaluated in this article, or claim that may be made by its manufacturer, is not guaranteed or endorsed by the publisher.

## Footnotes

1
http://drive5.com/uparse/

2
http://rdp.cme.msu.edu/

## Supplementary Material

Supplementary Figure 1 Two yield indicators, **(A)** grain and **(B)** straw, during maize harvest stage under P125 (with a rate of 125 kg P_2_O_5_ ha^–1^) and P90 (with a rate of 90 kg P_2_O_5_ ha^–1^). Significances between the two treatments were compared using one-way *t*-test (0.01 < *p* ≤ 0.05 = ^∗^).

Supplementary Figure 2 Rank Abundance curve of bacterial and fungal community. **(A)** bacteria community; **(B)** fungi community. Different colored curves represent different samples, and its smoothness indicates the diversity of species.

Supplementary Figure 3 Species differences of bacterial communities at the phylum **(A)** and genus **(B)** level. Species with significant differences at the phylum and levels using Welch’s *t*-test (*p* < 0.05). P125, with a rate of 125 kg P_2_O_5_ ha^–1^; P90, with a rate of 90 kg P_2_O_5_ ha^–1^; S, seedling stage; E, ear stage; F, flowering stage; and H, harvest stage.

Supplementary Figure 4 Species differences of fungal communities at the phylum **(A)** and genus **(B)** level. Species with significant differences at the phylum and genus levels using Welch’s *t*-test (*p* < 0.05). P125, with a rate of 125 kg P_2_O_5_ ha^–1^; P90, with a rate of 90 kg P_2_O_5_ ha^–1^; E, ear stage; F, flowering stage; and H, harvest stage.

Supplementary Figure 5 Heatmaps of bacterial community composition. The color gradient shows the changes in the abundance of different species in the sample at the phylum **(A,B)** and genus **(C,D)** levels. The legend on the right is the *lg* value, and species with significant differences are analyzed by one-way ANOVA (0.01 < *p* ≤ 0.05 = ^∗^, 0.001 < *p* ≤ 0.01 = ^∗∗^). P125, with a rate of 125 kg P_2_O_5_ ha^–1^; P90, with a rate of 90 kg P_2_O_5_ ha^–1^; S, seedling stage; E, ear stage; F, flowering stage; and H, harvest stage.

Supplementary Figure 6 Heatmaps of fungal community composition. The color gradient shows the changes in the abundance of different species in the sample at the phylum **(A,B)** and genus **(C,D)** level. The legend on the right is the *lg* value, and species with significant differences are analyzed by one-way ANOVA (0.01 < *p* ≤ 0.05 = ^∗^, 0.001 < *p* ≤ 0.01 = ^∗∗^). P125, with a rate of 125 kg P_2_O_5_ ha^–1^; P90, with a rate of 90 kg P_2_O_5_ ha^–1^; S, seedling stage; E, ear stage; F, flowering stage; and H, harvest stage.

Supplementary Table 1 PCR primers for 16S rRNA and ITS in this work.

Supplementary Table 2 PCR amplification process of 16S rRNA gene and ITS gene.

Supplementary Table 3 Correlation coefficient between environmental factors and microbial structure.

Supplementary Table 4 Bacterial network structure properties.

Supplementary Table 5 Fungal network structure properties.
