# Supplementary figures and images for: Rhizosphere Microbial Communities Are Significantly Affected by Optimized Phosphorus Management in a Slope Farming System
Source: Front Microbiol. 2021 Sep 13;12:739844. doi: 10.3389/fmicb.2021.739844 (PMC8473901; doi:10.3389/fmicb.2021.739844)

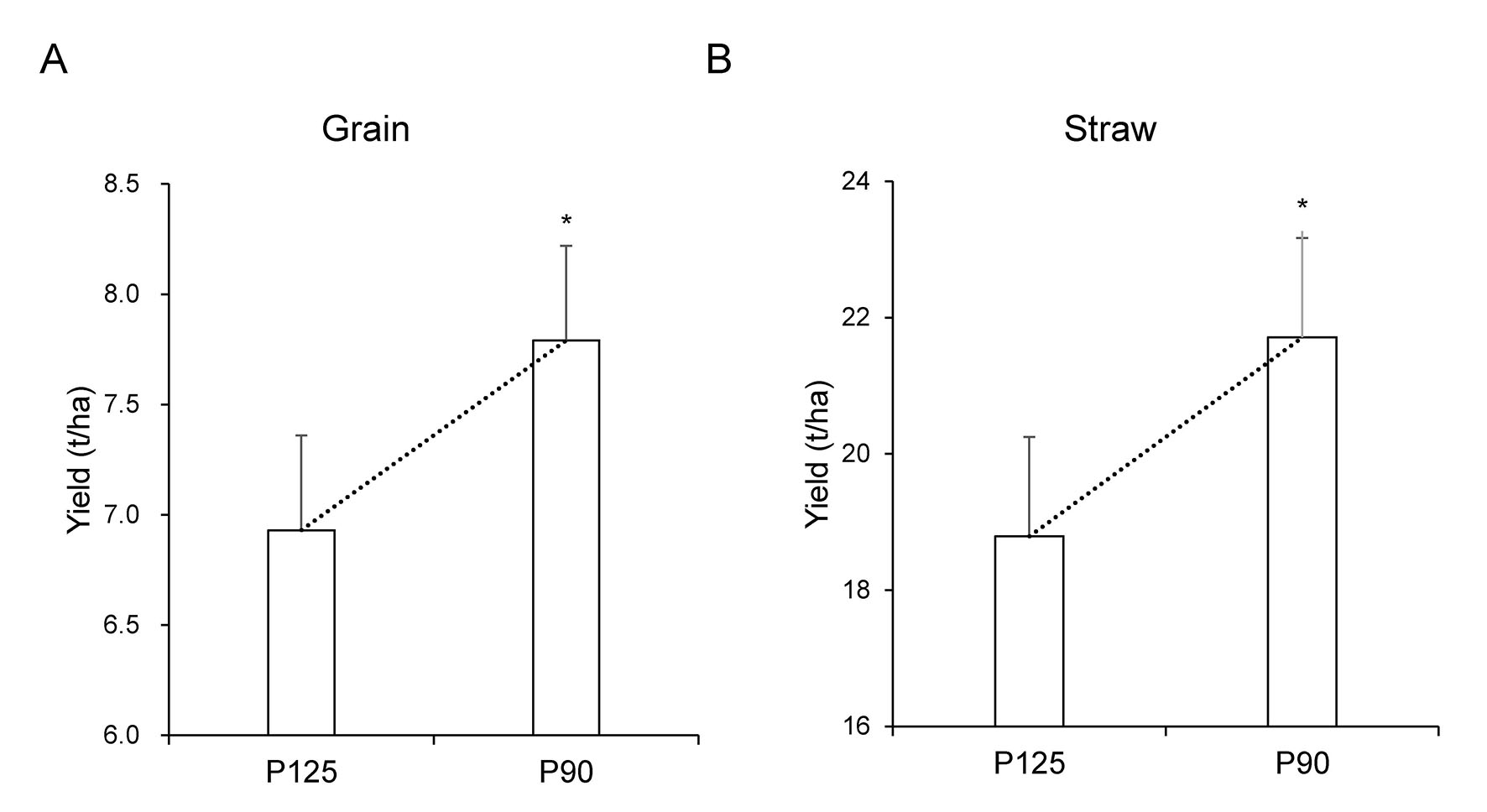

Supplement: Supplementary Figure 1 — Two yield indicators, (A) grain and (B) straw, during maize harvest stage under P125 (with a rate of 125 kg P2O5 ha–1) and P90 (with a rate of 90 kg P2O5 ha–1). Significances between the two treatments were compared using one-way t-test (0.01 < p ≤ 0.05 = ∗). [file Image_1.JPEG]

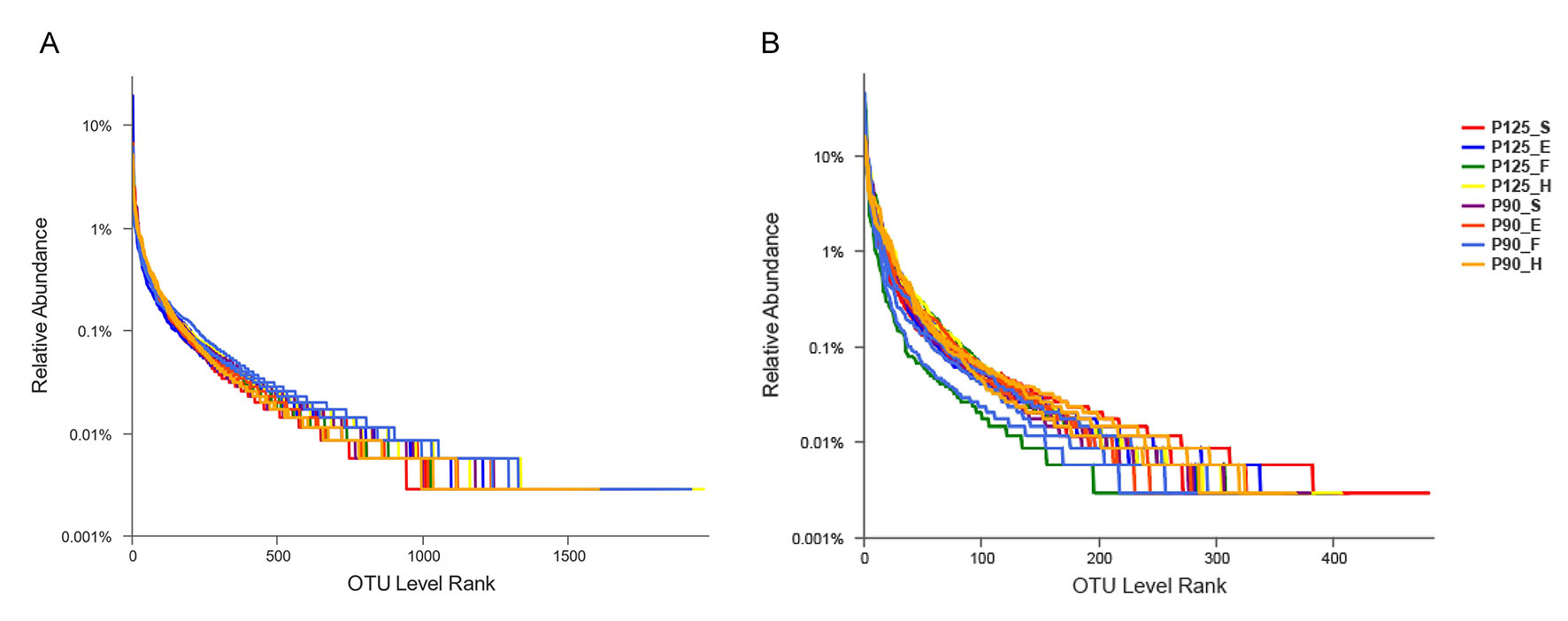

Supplement: Supplementary Figure 2 — Rank Abundance curve of bacterial and fungal community. (A) bacteria community; (B) fungi community. Different colored curves represent different samples, and its smoothness indicates the diversity of species. [file Image_2.JPEG]

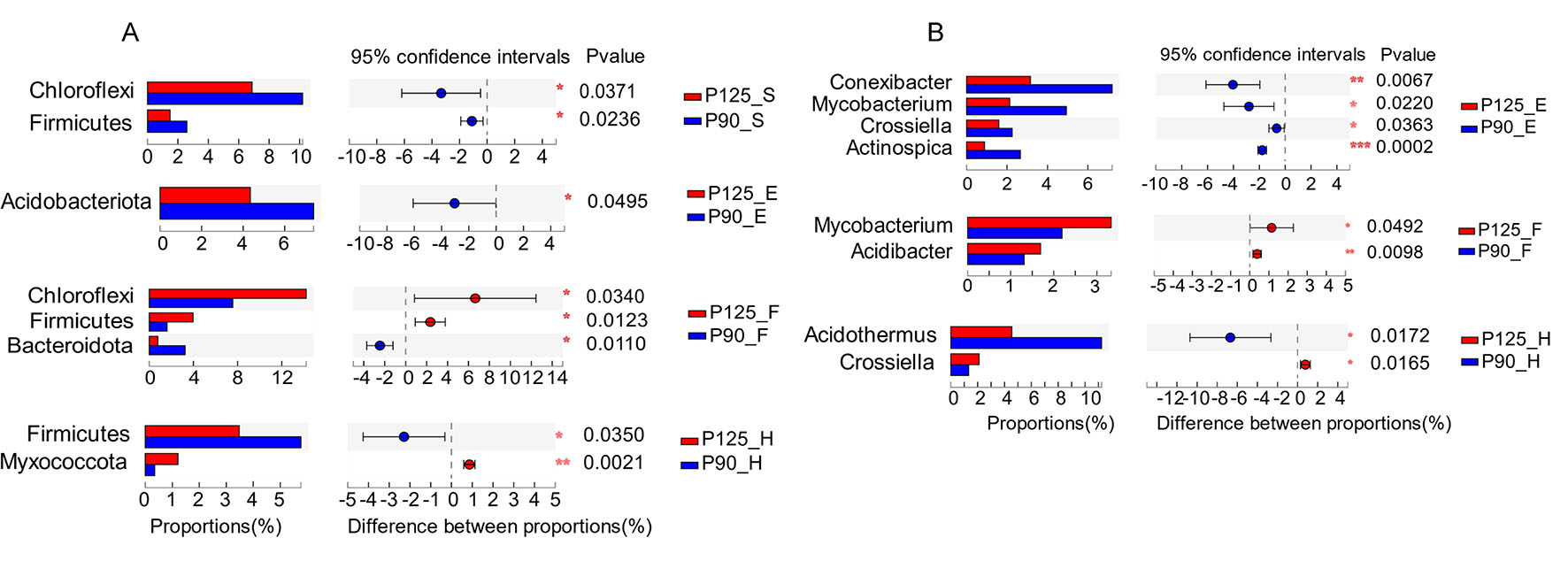

Supplement: Supplementary Figure 3 — Species differences of bacterial communities at the phylum (A) and genus (B) level. Species with significant differences at the phylum and levels using Welch’s t-test (p < 0.05). P125, with a rate of 125 kg P2O5 ha–1; P90, with a rate of 90 kg P2O5 ha–1; S, seedling stage; E, ear stage; F, flowering stage; and H, harvest stage. [file Image_3.JPEG]

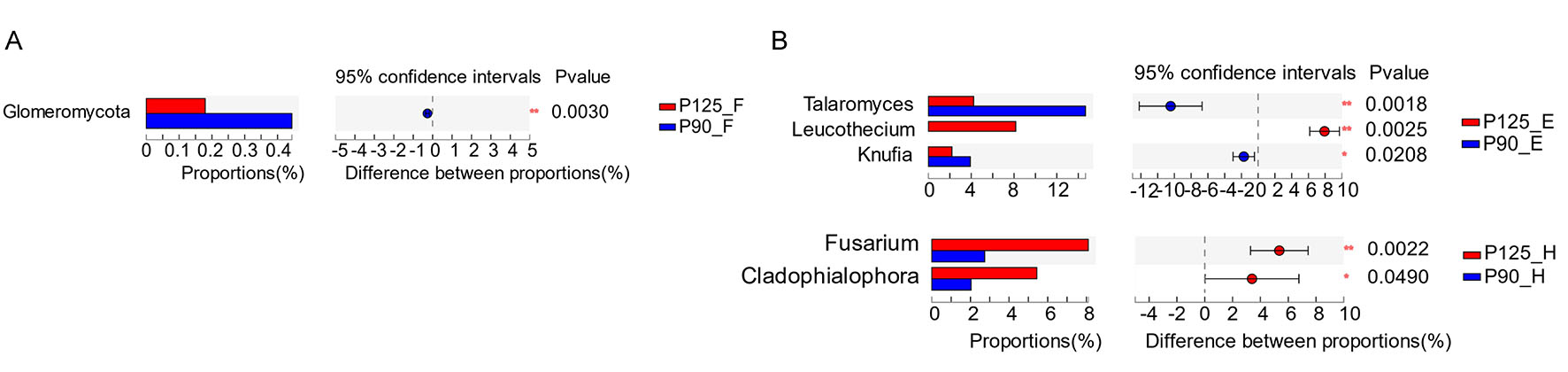

Supplement: Supplementary Figure 4 — Species differences of fungal communities at the phylum (A) and genus (B) level. Species with significant differences at the phylum and genus levels using Welch’s t-test (p < 0.05). P125, with a rate of 125 kg P2O5 ha–1; P90, with a rate of 90 kg P2O5 ha–1; E, ear stage; F, flowering stage; and H, harvest stage. [file Image_4.JPEG]

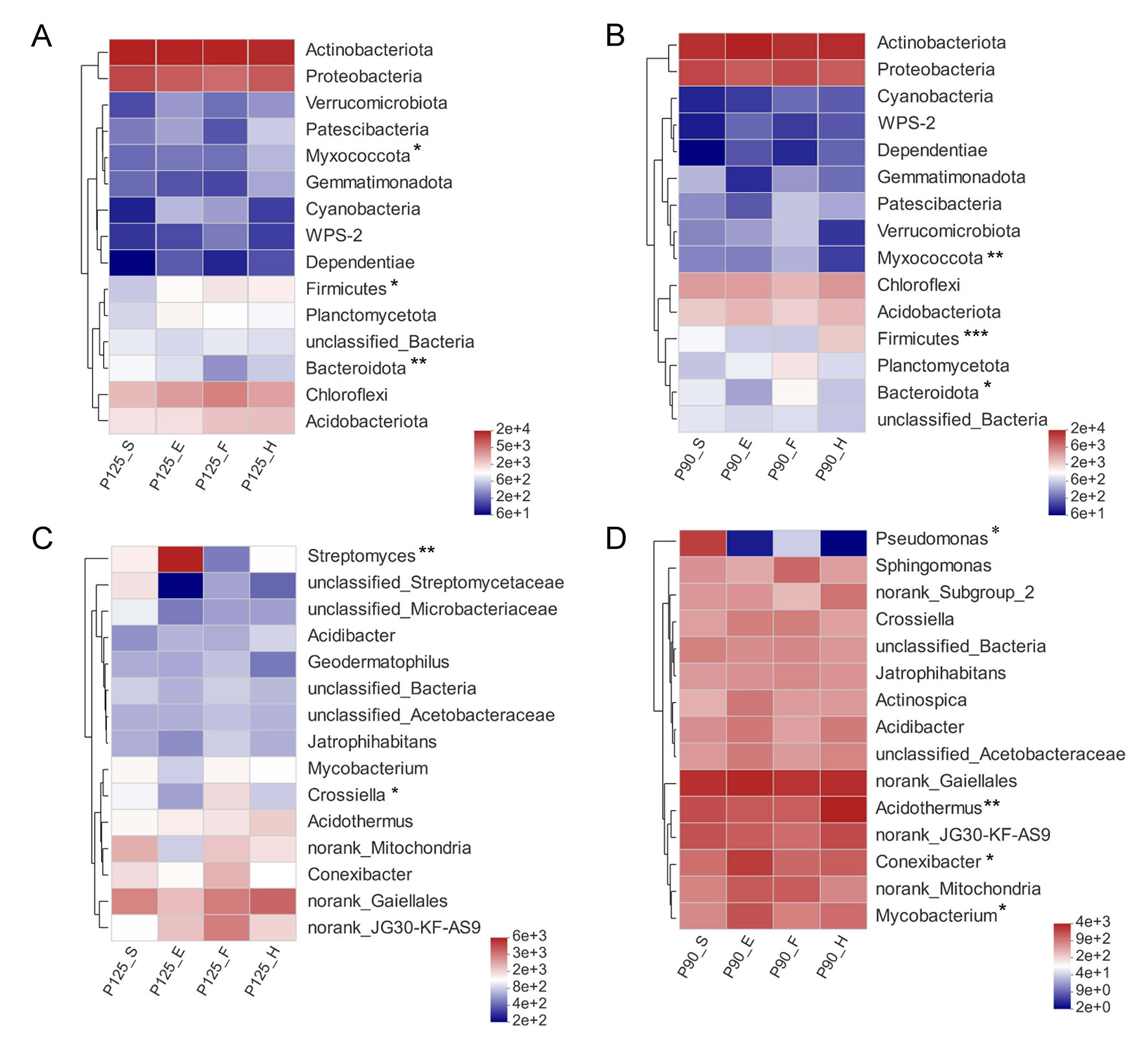

Supplement: Supplementary Figure 5 — Heatmaps of bacterial community composition. The color gradient shows the changes in the abundance of different species in the sample at the phylum (A,B) and genus (C,D) levels. The legend on the right is the lg value, and species with significant differences are analyzed by one-way ANOVA (0.01 < p ≤ 0.05 = ∗, 0.001 < p ≤ 0.01 = ∗∗). P125, with a rate of 125 kg P2O5 ha–1; P90, with a rate of 90 kg P2O5 ha–1; S, seedling stage; E, ear stage; F, flowering stage; and H, harvest stage. [file Image_5.JPEG]

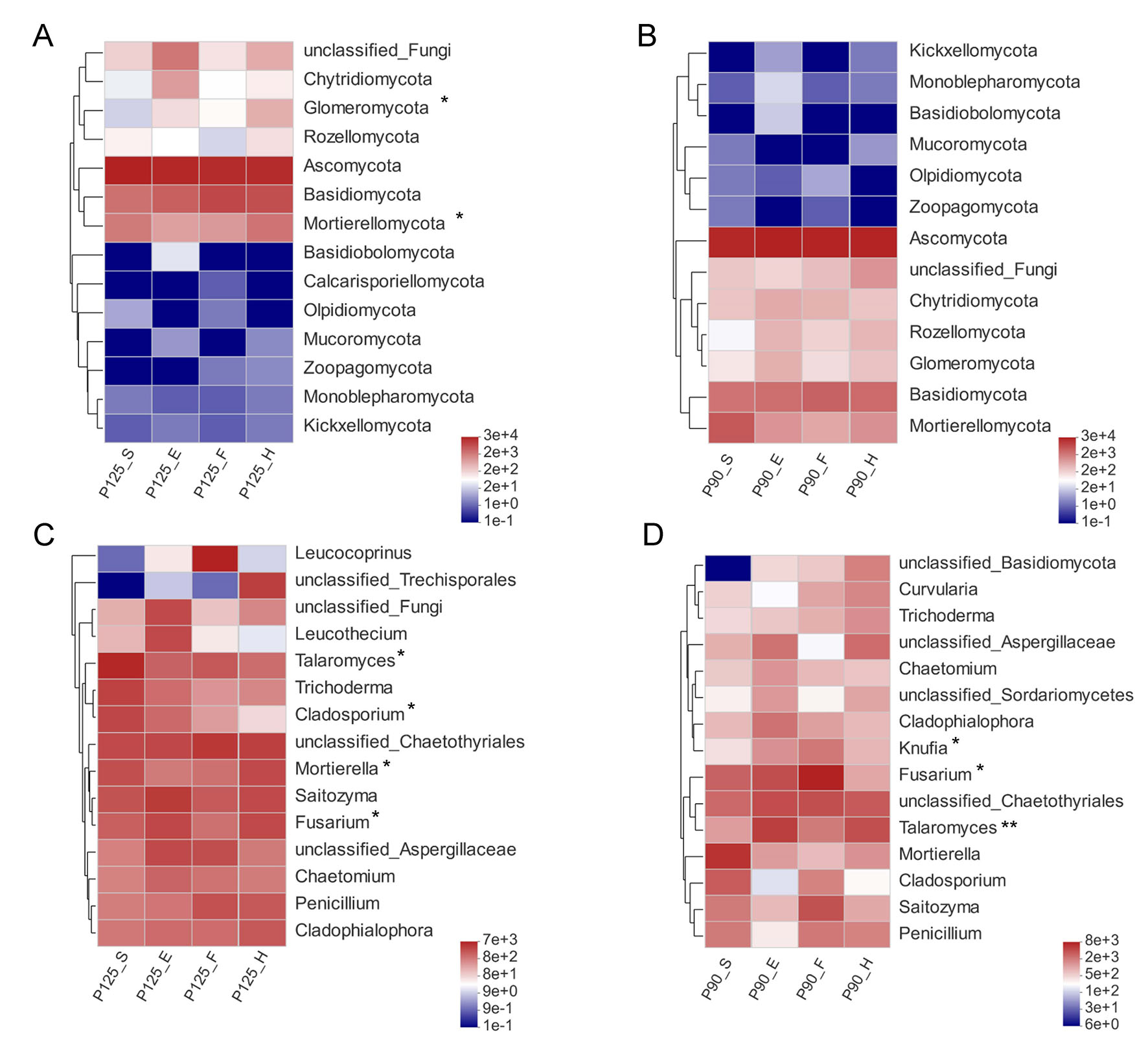

Supplement: Supplementary Figure 6 — Heatmaps of fungal community composition. The color gradient shows the changes in the abundance of different species in the sample at the phylum (A,B) and genus (C,D) level. The legend on the right is the lg value, and species with significant differences are analyzed by one-way ANOVA (0.01 < p ≤ 0.05 = ∗, 0.001 < p ≤ 0.01 = ∗∗). P125, with a rate of 125 kg P2O5 ha–1; P90, with a rate of 90 kg P2O5 ha–1; S, seedling stage; E, ear stage; F, flowering stage; and H, harvest stage. [file Image_6.JPEG]
